# Multi-method, longitudinal documentation of adaptations and their impact in the Accelerating Colorectal Cancer Screening and Follow-up through Implementation Science San Diego colorectal cancer screening implementation study

**DOI:** 10.1093/tbm/ibag072

**Published:** 2026-10-06

**Authors:** Valesca Largaespada, Jesse N Nodora, Jennifer Covin, Yesenia Preciado-Hidalgo, Kristine Ortwine, Nicole Howard, Samir Gupta, Maria Elena Martinez, Scott C Roesch, Sheila Castaneda, Borsika A Rabin

**Affiliations:** UC San Diego Moores Cancer Center, University of California San Diego, La Jolla, CA, United States; UC San Diego Moores Cancer Center, University of California San Diego, La Jolla, CA, United States; Department of Radiation Medicine and Applied Sciences, UC San Diego School of Medicine, University of California San Diego, La Jolla, CA, United States; Health Quality Partners of Southern California, San Diego, CA, United States; Health Quality Partners of Southern California, San Diego, CA, United States; Integrated Health Partners of Southern California, San Diego, CA, United States; Health Quality Partners of Southern California, San Diego, CA, United States; UC San Diego Moores Cancer Center, University of California San Diego, La Jolla, CA, United States; Veterans Affairs San Diego Healthcare System, La Jolla, CA, United States; UC San Diego Moores Cancer Center, University of California San Diego, La Jolla, CA, United States; Herbert Wertheim School of Public Health and Human Longevity Science, University of California San Diego, La Jolla, CA, United States; Department of Psychology, San Diego State University, San Diego, CA, United States; Department of Psychology, San Diego State University, San Diego, CA, United States; Herbert Wertheim School of Public Health and Human Longevity Science, University of California San Diego, La Jolla, CA, United States; Altman Clinical and Translational Research Institute Dissemination and Implementation Science Center, University of California San Diego, La Jolla, CA, United States

**Keywords:** adaptations, implementation science, colorectal cancer screening, RE-AIM framework

## Abstract

**Background:**

Adaptations are common in implementation studies, yet they are rarely documented systematically. Understanding when, why, and how adaptations occur can improve implementation processes, support interpretation of study outcomes, and inform scaling in diverse settings. Using comprehensive frameworks such as reach, effectiveness, adoption, implementation, and maintenance (RE-AIM) and expanded framework for reporting adaptations and modifications-enhanced can guide systematic documentation, but practical, standardized approaches remain underdeveloped.

**Purpose:**

This study describes a multi-method, framework-guided approach to documenting adaptations across all phases of the accelerating colorectal cancer screening and follow-up through implementation science San Diego study, which implemented multilevel, evidence-based interventions to reduce colorectal cancer in community health center (CHC) systems.

**Results:**

We documented 44 adaptations over the pre-implementation (*n* = 22), implementation (*n* = 19), and sustainment (*n* = 3) phases. Most were verified through emails/meetings, with research staff/CHC staff as primary informants. Adaptations most frequently involved intervention materials, content, and format, and were primarily driven by pragmatic considerations to align with CHC workflows, patient needs, and contextual factors. While most adaptations had no major impact on RE-AIM outcomes, several improved implementations (e.g. centralizing FIT kit procurement to address COVID-19-related supply chain issues). Negative impacts were rare.

**Conclusions:**

A multi-method, framework-based approach to adaptation documentation is feasible and valuable in complex, real-world implementation studies. Early and ongoing tracking supports proactive problem-solving, enhances contextual fit, and informs sustainability planning. This methodology can serve as a model for systematic adaptation tracking in safety-net and community-based healthcare settings, strengthening the evidence base for how adaptations influence implementation and effectiveness outcomes.

**Clinical trials registration:**

The Clinical Trials Registration #NCT04941300.

## Teaser

Careful tracking of program changes helped community health centers overcome challenges and keep a cancer screening program running effectively. This process can guide other programs to adapt and succeed in real-world conditions.

Implications **Practice:** Practitioners implementing multilevel, evidence-based interventions in community health center and safety-net settings should anticipate and plan for adaptation, particularly during pre-implementation, and can be reassured that thoughtfully managed adaptations are unlikely to compromise, and may even improve, implementation and effectiveness outcomes.

**Policy:** Funders and health system leaders should incentivize and resource practical, low-burden adaptation tracking, such as through data infrastructure support and protected staff time, as a standard component of implementation initiatives in community-based and safety-net healthcare systems.

**Research:** Future studies should build on this multi-method, framework-guided approach to develop more objective, longitudinal methods for assessing how adaptations differentially affect implementation and effectiveness outcomes across study sites and phases.

## Introduction

Although adaptations are common in implementation studies, they are rarely documented in a systematic manner [1]. Gaining a comprehensive understanding of when adaptations occur and why they are made throughout the study lifespan can help refine implementation processes, enhance interpretation of study outcomes, and inform future scaling efforts in diverse settings. They can also inform the balance between needed adaptations and fidelity [2]. Comprehensive frameworks exist to guide the process of adaptations [3, 4], but standardized approaches for capturing and analyzing adaptations are often lacking [5].

There is a growing body of literature examining the value and methodology to document adaptations, including prominent frameworks available for systematic documentation, guidelines and linked literature on what and how should be documented by whom, when, and how frequently [1, 6–9]. A more recent publication also discusses the challenges and strategies for assessing the impact of adaptations specifically suggesting a proactive approach to assessment of impact based on the key research questions relevant to the study [7]. Many questions remain regarding the most pragmatic, yet comprehensive way, to capture adaptations in complex real-world studies.

This paper focuses on adaptation documentation, describing a multi-method approach used to capture and characterize adaptations during a pragmatic colorectal cancer (CRC) prevention study in San Diego and highlighting its methodological for implementation science. Funded by the National Cancer Institute’s Accelerating Colorectal Cancer Screening and Follow-Up Through Implementation Science San Diego study (ACCSIS) Cancer Moonshot℠ initiative, the program aims to evaluate the implementation and scalability of evidence-based, multilevel interventions (EBIs) to reduce CRC disparities among underserved populations in the United States. Specifically, the San Diego ACCSIS pragmatic study focuses on developing regionally tailored solutions to support the adoption, implementation, and sustainability of a comprehensive approach to CRC screening and follow-up across community health center (CHC) systems. With an academic–community partnership approach, Health Quality Partners of Southern California (HQP)—part of the Health Center Partners of Southern California Family of Companies serves as the hub—and four participating CHC systems serve as spokes. In the hub and spoke organization design, the hub serves as the anchor establishment to offer broad services to secondary establishments named as spoke [10]. In San Diego County, the absence of a county hospital and limited direct provision of primary care services to medically underserved populations positions CHCs as the primary healthcare providers for the region’s safety-net population [11]. The study design is described in detail in a protocol paper [11].

### Conceptual framework and implementation science measures

Implementation and evaluation were guided by the practical robust implementation and sustainability model (PRISM). PRISM comprises a set of multilevel, multi-perspective contextual domains—including the intervention, recipients, implementation and sustainability infrastructure, and external environment—alongside the widely used reach, effectiveness, adoption, implementation, and maintenance (RE-AIM) outcomes: reach, effectiveness, adoption, implementation, maintenance outcomes [12]. By adopting this methodology, sustainability and scalability were evaluated using the PRISM contextual domains of implementation and sustainability infrastructure and the external environment, in conjunction with the RE-AIM maintenance outcome. As such, the primary goal of this paper is to showcase how adaptations can be documented in a systematic manner in a complex implementation study.

## Methods

### Study context, intervention, and implementation strategy

The ACCSIS San Diego pragmatic study emerged from an academic-CHC partnership involving HQP, which serves as the “hub,” and four participating CHC systems, the “spokes.” One CHC system withdrew from the study prior to implementation. Any adaptations that impacted the CHC prior to withdrawal were documented. The pragmatic study employed a Hub-and-Spoke implementation model, in which HQP (the hub), offered centralized support to: (i) deliver mailed fecal immunochemical test (FIT) outreach and reminders; (ii) coordinate navigation for abnormal FIT follow-up and referral to care; and (iii) provide expert guidance on the implementation of evidence-based interventions (EBIs) to member CHC organizations (spokes), where patient care was delivered [11]. Adaptations were made to the intervention and implementation strategies (see Online Appendix 1 for a full list of adaptations). Adaptations that took place before and during implementation as compared to the original protocol of the study to improve fit of the program with the local context.

The interventions comprise an invitation primer, a mailed FIT with completion instructions, and phone and text-based reminders, as well as patient navigation protocol to promote colonoscopy completion after abnormal FIT. Implementation science measures are collected to assess acceptability, adaptations to both the intervention and usual care, and the sustainability of the intervention strategies [11]. A brief EBI workflow diagram linking EBI components to outcomes is included (see Online Appendix 2).

### Tracking methodology

Study team members with implementation science experience led the documentation and analysis of the adaptations, in collaboration with the broader research team and the hub. In this pragmatic study, adaptations are defined broadly to include any modifications to the intervention itself, implementation strategies, contextual factors, or research methods—including study design, measurement tools, recruitment procedures, and inclusion/exclusion criteria. We present adaptations occurring at both the hub and spoke levels, within participating CHCs, and across core elements of the study such as the mailed FIT outreach process, care coordination protocols, and research logistics, including Institutional Review Board (IRB) processes, data collection, and analysis procedures. The documentation of adaptations was guided by the reach, effectiveness, adoption, implementation, and maintenance (RE-AIM) expanded framework for reporting adaptations and modifications-enhanced (FRAME) framework [9] following methodology described by Rabin *et al.* [13]. There were 18 key characteristics documented about each adaptation via a tracking tool (Online Appendix 3). For example, for the characteristics of type of change, the answer options included: (i) tailoring to individuals; (ii) adding a component; (iii) removing a component; (iv) condensing a component; (v) extending a component; (vi) substituting a component; (vii) changing the order of components; (viii) integrating with other ongoing programs; (ix) repeating a component; (x) loosening the structure or protocol; and (xi) other modifications to the intervention. In addition to the characteristics of the adaptations, we also documented their impact. While multiple categories could be selected for each characteristic, we tried to limit selections to the best fitting top one to two answer options.

In order to systematically track adaptations, we utilized multiple key sources that served several purposes, including informing specific adaptations, identifying the timing of each adaptation, and determining the component affected. We employed the following documentation methods: (i) real-time centralized tracking database developed in REDCap; (ii) reminder emails disseminated to all research team members, including the hub; (iii) review of relevant study documents (e.g. study team meeting minutes, Brainwriting exercise notes [14, 15], practical robust implementation sustainability model (PRISM) context assessment team discussion notes); (iv) structured interviews with key stakeholders; and (v) regular check-in meetings, scheduled biweekly, with each CHC to inquire about adaptations.

Adaptations were documented prospectively using a real-time tracking system developed in REDCap, guided by the extended RE-AIM expanded FRAME framework [9, 16] (see Online Appendix 3). The real time adaptation tracking system was refined over time through ongoing team discussions. As new adaptations were submitted, minor modifications and clarifications were made to improve consistency and specificity in documentation. Only the lead adaptation coordinator entered all adaptations. This process ensured the consistent use of the tracking system.

Biweekly reminder emails were sent to all team members to encourage the submission of adaptations via Word document, which were subsequently entered into the REDCap system by the adaptation lead. The reminder emails (see Online Appendix 4 for a template) were disseminated to all research team members, including the hub, by the implementation coordinator. An adaptation workgroup, consisted of the lead adaptation coordinator and the senior implementation scientist, was formed and met monthly to review the tracking process and discuss reported adaptations.

We also conducted a retrospective review of study materials—including meeting minutes, reports, and internal logs—to identify adaptations that occurred prior to the establishment of the workgroup and real-time tracking system. These data were entered into the REDCap database. Research team members were invited to review the full list of documented adaptations to ensure their completeness and accuracy.

Quarterly reviews of study documents (e.g. study team meeting minutes, Brainwriting exercise notes, PRISM context assessment team discussion notes, and decision tracking logs maintain to summarize decisions made throughout the entirety of the study) were conducted to identify any adaptations that may have been missed during real-time tracking.

Structured interviews (see Online Appendix 5 for interview guide excerpts) were conducted with key stakeholders at participating CHCs, including lead study staff, providers, and CHC leaders with knowledge of local operations, organizational priorities, and familiarity with the ACCSIS pragmatic study. Interviews were carried out at two time points: during implementation—defined as the period when mailed FIT outreach was delivered to eligible patients and care coordination was provided for those with abnormal FIT results—and post-implementation, which refers to the period following the final wave of mailed FIT outreach. Stakeholders were asked to describe implementation challenges and any resulting adaptations.

All interviews were conducted virtually and recorded, with transcription performed by a third-party vendor. A rapid qualitative analysis was used to code interview data, which were then incorporated into the adaptation tracking database. These adaptations will be further analyzed using a multi methods approach to describe the number, type, nature, and impact of adaptations across the study period.

In order to mitigate forgetfulness and attempt to capture all adaptations in a timely manner, regular meeting check-ins were conducted to facilitate identification of adaptations and to verify whether any previously undocumented adaptations had occurred, supporting coding validation. These check-ins took place during study team meetings throughout the implementation phase.

Additionally, biweekly meetings were held with each participating CHC to systematically inquire about potential adaptations. The following guiding questions were used during these discussions: (i) How are things going? (ii) Have there been any changes to the protocol? (iii) Have you experienced any challenges?

### Structured reflection on adaptation impact

Once the study concluded, the impact of adaptations was assessed retrospectively by a structured team-based reflection process comprising of the lead adaptation coordinator and a member of the hub, who were more closely familiar with the adaptations. The process involved: (i) review of all 44 documented adaptations by the adaptation coordinator and hub member; (ii) consultation with the broader research team; (iii) triangulation with available quantitative and qualitative data where present; and (iv) structured deliberation where data were absent. Additional input was solicited from the research team and the hub. When available, data were used to inform the assessment of impact. In the absence of relevant data, the team relied on collective pragmatic experience and deliberation to determine the impact. Although imperfect, this methodology provides a documented and replicable process for assessing adaptation impacts, addressing ongoing methodological challenge in the field and aligning with current guidance from Aschbrenner *et al.* [7].

### Data management and analysis

All adaptations regardless of their source were entered into the dedicated adaptation database in REDCap. The full research team conducted reviews of the database to ensure data integrity, including identification of missing or duplicate adaptation entries. One previously undocumented adaptation was identified during this process. In addition, adaptations initially marked “too early to know” regarding their impact were revisited and updated as appropriate. After cleaning and organizing the adaptation data, frequency tables and summary counts were used to organize them by the key adaptation characteristics. For some variables, multiple response options could be selected. For analyses presented in Tables 1, 2, 4, and 5, multiple response options selected for a single adaptation were counted under each applicable category.

**Table 1:** Adaptations by data source and study phase.**^a^**

Data source	Pre-implementation	Implementation	Sustainment	Total
Email	5	2	0	7
Meeting	9	3	1	13
Other	1	1	0	2
Emails, meetings	6	13	2	21
Email, phone	1	0	0	1
Care coordination protocol	0	1	0	1

a Multiple adaptations had more than one source.

**Table 2:** Adaptation information source by study phase**.^a^**

Data source	Pre-implementation	Implementation	Sustainment	Total
Research staff	13	5	1	19
HQP	5	9	2	16
Physician	0	0	0	0
Clinic coordinator	2	0	0	2
Lab manager	0	0	0	0
Third-party vendor	0	2	0	2
Other	2	7	2	11

a Multiple adaptations had more than one source.

## Results

Adaptations were primarily identified and verified through routine emails and meetings. Most adaptations focused on modifying intervention materials, content, or delivery format to better align with CHC workflows, patient needs, and changing contextual circumstances. Overall, adaptations were largely pragmatic and intended to facilitate implementation rather than alter core intervention components. Consistent with this goal, most adaptations had little discernible effect on RE-AIM outcomes, although several enhanced implementation processes—e.g. centralizing FIT kit procurement helped mitigate COVID-19-related supply chain disruptions and supported program continuity. Adaptations perceived to have negative consequences were uncommon, suggesting that ongoing tailoring generally strengthened implementation while preserving intervention integrity.

### Timing of adaptations

We organized adaptations by three distinct timepoints in the study timeline and distinguished among pre-implementation, implementation, and sustainment phases. Most adaptations were identified in pre-implementation (*n* = 22), followed by the implementation (*n* = 19), and sustainment (*n* = 3) phases.

### Data sources for adaptations

Table 1 presents the distribution of adaptations captured across various data sources. The majority of adaptations were documented through emails and meetings, collectively accounting for 21 adaptations. Additionally, meetings served as a primary documentation method, capturing 13 adaptations, while emails recorded seven adaptations. There were 22 adaptations that were captured by multiple methods.

### Position of informant

Table 2 illustrates the distribution of adaptations recorded by various informants. Research staff documented the highest number of adaptations (*n* = 19), followed closely by HQP (*n* = 16). The implementation phase accounted for the highest number of adaptations submitted by informants, totaling 23 adaptations.

### Types of adaptation

Guided by the RE-AIM expanded FRAME [13] and the PRISM [12, 17], adaptations were characterized using key adaptation constructs. These constructs included: (i) documentation of the adaptation, (ii) the individual or group responsible for the change, (iii) the nature of the modification, (iv) the study component most affected, (v) the rationale for the adaptation, and (vi) whether the change was influenced by the COVID-19 pandemic.

The distribution of the adaptations across sites and timepoints—pre-implementation, implementation, and sustainment—is presented in Table 3. The majority of the adaptations (*n* = 22) occurred during the pre-implementation phase, followed by 19 adaptations during the implementation phase, and only three adaptations during the sustainment phase. The most frequent site of adaptation was in the non-CHC (non-CHC) settings, where 11 adaptations were documented during the pre-implementation phase. There were 12 adaptations that were captured by multiple methods.

**Table 3:** Number of adaptations by study sites and phase.

Sites	Study phase		
	Pre-implementation	Implementation	Sustainment
CHC 1	1	3	0
CHC 2	3	1	0
CHC 3	0	1	0
CHC 4	1	5	0
CHC 1, CHC 4	1	3	2
CHC 1, CHC 2, CHC 3, CHC 4	5	0	0
CHC 1, CHC 2, CHC 4	0	2	0
Non-CHC adaptations	11	4	1
Total	22	19	3

### Adaptation elements (Table 4)

The most commonly categorized adaptation element was “Other” (*n* = 19), which encompassed modifications related to the intervention materials and content, as well as the format (*n* = 9), study design, documentation, and assessment (*n* = 4), and changes to the broader study context (*n* = 6). Format was the second most frequently categorized adaptation element, with the majority of these adaptations occurring during the pre-implementation phase of the study. Many of these adaptations occurred during the pre-implementation phase (*n* = 23). There were 22 adaptations that were captured by multiple methods. Notably, certain adaptations affected multiple CHCs while others were limited to a subset or a single CHC.

**Table 4:** Adaptation constructs across study phase.

Adaptation constructs	Pre-implementation	Implementation	Sustainment	Total
**Elements** ^a^				
Setting	3	3	0	6
Format	4	3	0	7
Personnel involved	1	5	0	6
Target population	1	2	0	3
Intervention presentation	2	3	0	5
Others	11	5	3	19
Intervention material content and format	8	1	0	9
Study design, documentation, and assessment	1	1	2	4
Change to context	2	3	1	6
**What was changed** ^a^				
Tailoring to individuals	0	0	0	0
Adding a component	0	0	0	0
Removing a component	0	1	0	1
Condensing a component	1	0	0	1
Extending a component	2	1	0	3
Substituting a component	2	1	0	3
Changing the order of components	0	1	0	1
Integrating with other programs we are doing	1	1	0	2
Repeating a component	0	0	0	0
Loosening the structure or protocol	4	0	0	4
Otherwise changing the intervention	7	7	0	14
How the intervention is delivered in the study^b^	3	4	1	8
Study related changes^b^	2	4	2	8
**Who was responsible for this change** ^a^				
Entire or most of the team	8	3	0	11
Research team	4	4	1	9
HQP	4	3	1	8
CHC or individual clinic	9	8	1	18
FIT vendor^b^	0	3	0	3
**How or on what basis were these changes made** ^a^				
Based on our mission or values	0	1	0	1
Based on a framework	0	0	0	0
Based on our knowledge or experience of working with patients	4	3	0	7
Based on patient knowledge or experiences	0	0	0	0
Based on QI data, summary information or results	2	3	1	6
Based on pragmatic/practical considerations	9	11	2	22
Based on financial incentives/payment	0	0	0	0
Based on feedback or suggestions	6	2	0	8
Additional funds availability to increase intensity^b^	1	0	0	1
Error^b^	0	3	0	3
**Why was the change made** ^a^				
To increase the number or type of patients contacted (reach)	0	2	0	2
To enhance the impact or success of the intervention for all or important subgroups (effectiveness)	7	3	0	10
To make it possible to involve more teams, team members or staff (adoption)	0	1	0	1
To make the intervention delivered more consistently; to better fit the CHC/clinic, clinician needs, patients flow or EHR; for practical reasons (implementation)	11	10	0	21
To institutionalize or sustain the intervention (maintenance)	0	1	0	1
To respond to external pressures or policy	4	2	2	8
To save money or other resources (implementation)	2	0	0	2
To respond to internal change (i.e. staff change, site closures, CHC preference, etc.)^b^	4	5	2	11
**Adaptation due to COVID-19 pandemic**				
Yes	5	1	1	7

a Most adaptations had multiple responses in this construct.

b Emerged as a novel theme based on responses and reflects informed consensus of the study team rather than independent outcome measurement.

### Type of change

Another key area of adaptation was the type of change that occurred. Modifications to the intervention itself accounted for the majority of adaptations (*n* = 14). The second most common categories were changes in how the intervention was delivered and study-related modifications, each accounting for eight adaptations. The majority of these changes occurred during the pre-implementation phase (*n* = 22), emphasizing the focus on refining the intervention before full-scale implementation to enhance feasibility and effectiveness.

### Who was responsible for this change?

In most cases, the primary responsibility for adaptations fell on the CHC or individual clinic (*n* = 18). The second most common entity responsible for adaptations was the entire study team or a majority of its members (*n* = 11). The pre-implementation phase accounted for the majority of the adaptations (*n* = 25).

### How or on what basis were these changes made?

The majority of adaptations were driven by pragmatic or practical considerations (*n* = 22). The pre-implementation phase accounted for the highest number of modifications (*n* = 25), emphasizing the importance of early-stage adjustments to optimize intervention feasibility and implementation. Two novel themes emerged as a result of responses, additional funds availability to increase intensity (*n* = 1) and error (*n* = 3). Error emerged as a novel theme based on responses. All three errors consisted of oversights made by the third-party vendor used to deliver the mailed FIT outreach intervention.

### Why was this change made?

The most commonly reported reason for adaptation was to enhance the consistency of intervention delivery and to better align with the needs of the CHC/clinic, clinicians, patient flow, or electronic health record (EHR) systems for practical implementation purposes (*n* = 21). The highest number of adaptations occurred during the pre-implementation phase (*n* = 11), reinforcing the importance of early modifications in optimizing intervention feasibility. Additionally, a novel theme, Response to Internal Change, emerged from the data (*n* = 11). In two instances, the internal change was related to staff resignations at the CHCs.

### COVID-19 impact

The majority of adaptations were not driven by the impact of the COVID-19 pandemic, suggesting that modifications were primarily influenced by other contextual or practical implementation factors rather than external public health crises. An adaptation that was due to the COVID-19 pandemic was specific to a decision by HQP leadership to move to a permanent remote working environment. As a result, a PO box was established to allow for return of mailed FIT intervention materials.

### Study components impacted (Table 5)

The majority of the adaptations impacted the mailed FIT process (*n* = 25). Impacts to the mailed FIT process included a revised reminder schedule for patients to return FIT kits. An additional text message was included in the protocol. Originally, the reminder call schedule included two automated phone calls and one text message. Additionally, study material was reviewed by the CHCs. Feedback was obtained and revisions were reviewed by the MPIs. Any modifications made to the study material were noted as adaptations. The care coordination process, change in personnel, and change in participating sites were all affected equally (*n* = 5) by adaptations. There were four adaptations that impacted multiple study components. Due to operational changes in one CHC, some clinics sites were impacted. A clinic retired as primary care location and a new clinic site opened. Data management impacted four adaptations. In our study, data management is defined by how the data was obtained, preparing and identifying eligible patients, cleaning data, and revisions to data collection protocols.

**Table 5:** Study components impacted by adaptations.**^a^**

Study component	# of adaptations impacted
Care coordination process	5
Mailed FIT process	25
Usual care assessment protocol	2
Data management	4
Change in personnel	5
Change in participating sites	5

a Multiple adaptations had more than one source.

### Impact of adaptations

Figure 1 presents the influence of documented adaptations on implementation and effectiveness outcomes, as evaluated using the restructured RE-AIM framework. Each adaptation was categorized based on its observed impact: increased, decreased, no major change, unknown, or not applicable. The majority of adaptations (*n* = 22) were determined to have no substantial effect across RE-AIM domains.

**Figure 1:**
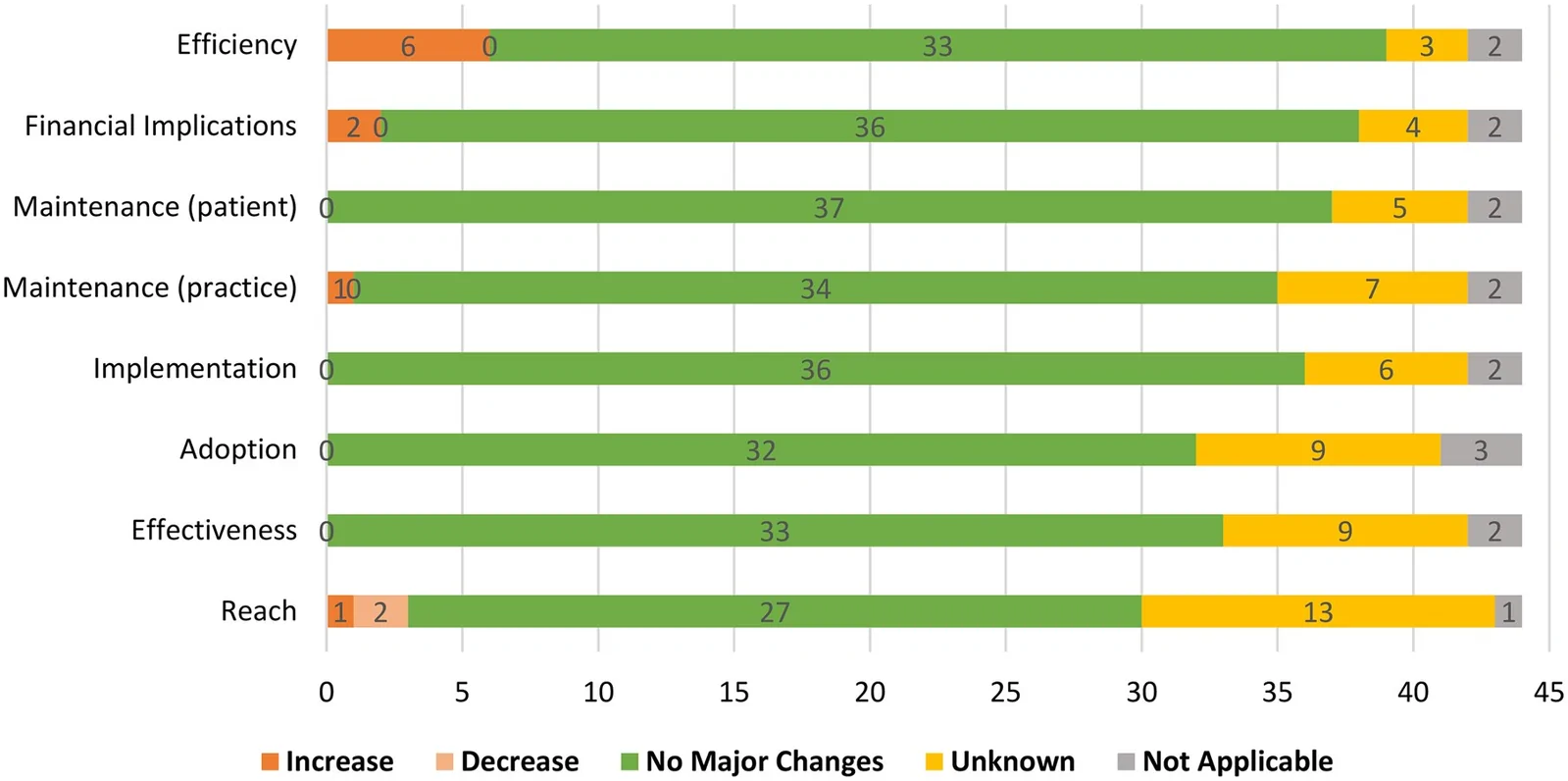
Impact of adaptation.

Seven adaptations were associated with increased improvements in outcomes. One notable example of increased outcome is related to the COVID-19 pandemic. The participating CHCs reported significant challenges in securing FIT kits. The challenges were largely attributed to FIT inventory issues at the contract laboratories that supply FIT and analyze and provide results to the CHC. Disruptions in their supply chain from manufactures of the FIT during the COVID pandemic impacted the routine workflow of the CHC in obtaining FIT supply for the study. In response, HQP identified and implemented a key operational adaptation: partnering with CNECT, a Group Purchasing Organization, to identify key contacts at a contracting laboratory to secure the FIT kits directly, and in bulk, from the laboratories instead of through pre-determined quantities and workflow of the individual health centers during supply disruptions. This adaptation was a change in the clinical and operational workflow at the CHCs related to obtaining FIT. By shifting the procurement responsibility from the CHCs to CNECT, the FIT kits were secured more reliably and efficiently.

Very few adaptations (*n* = 4) were associated with negative outcomes (two in reach and two in the financial implications domain), such as reduced performance. One notable example of a negatively rated adaptation on reach outcome was the withdrawal of two participating health center systems from the study due to sustainability concerns and internal priorities.

### Unknown impact

Of the 44 documented adaptations, two yielded “unknown” responses across all impact variables due to limitations in assessing their effects. The first adaptation occurred during the pre-implementation phase and pertained to the mailed FIT outreach intervention. Specifically, the reminder schedule was modified to include an additional text message, changing from two automated calls and one text message to two calls and two text messages. This change was intended to enhance intervention effectiveness and to optimize resource use. However, because the adaptation was made prior to implementation, it was not possible to determine its impact.

The second adaptation occurred during implementation and involved changes in the delivery of normal results letters. Initially, HQP was going to send letters to patients with normal as part of the study protocol, in addition to the CHC routine workflow of notifying their patient directly. The adaptation eliminated the duplicative process of sending normal results to patients. Although this adaptation was implemented, we were unable to determine its impact due to insufficient outcome data.

### Not applicable impact

One of the 44 documented adaptations was classified as “not applicable” across all impact variables. This adaptation was made based on pragmatic considerations during the pre-implementation phase of the mailed FIT outreach intervention. Specifically, a meeting was convened with participating CHCs to obtain approval for utilizing a third-party vendor to deliver the mailed FIT component. The meeting included a presentation by the vendor and participation from programmatic and compliance staff at the CHCs. All centers approved the collaboration between HQP and the third-party vendor. As this adaptation related to logistical planning and did not directly influence implementation or effectiveness outcomes, it was deemed not applicable for impact assessment.

## Discussion

This study demonstrates the value of systematically documenting adaptations across the lifespan of a multilevel implementation pragmatic study. We demonstrate how data can be collected in a pragmatic manner as well as sharing our findings in order to help build the science of adaptation. Using the RE-AIM expanded FRAME and multiple methods, we identified and documented 44 adaptations in real time and retrospectively, offering a comprehensive description of when, why, and how changes occurred during the pragmatic study. Our findings underscore the feasibility and utility of structured adaptation documentation in informing implementation efforts within complex community health settings.

Like the studies published by McCreight *et al.* [17] and McCarthy *et al.* [18], adaptations in our study were successfully captured across all phases of the study lifespan (i.e. pre-implementation, implementation, and sustainment). In our study, we found that most adaptations occurred during the pre-implementation phase (*n* = 22), highlighting the critical role of early-stage planning and refinement. This pattern reinforces the importance of proactive engagement with stakeholders to ensure feasibility and contextual fit prior to rolling out interventions. By addressing practical and logistical challenges up front (e.g. refining FIT outreach materials, adapting reminder schedules, or aligning workflows across hub and spoke organizations), researchers and implementers can facilitate the implementation process and related implementation outcomes.

We documented three adaptations in the sustainment phase. Continuing to document adaptations through the later (i.e. sustainment phase) can help in understanding what changes and approaches are needed for sustained delivery of the intervention versus initial implementation. Interestingly, unlike in our study where most adaptations were linked to the pre-implementation phase, a number of other studies that captured the timing of adaptations found that higher number of adaptations took place in the implementation phase than in the pre-implementation phase. For example, Reyes *et al.* [19], who observed the majority of the adaptations during the implementation phase, McCreight *et al.* [17], reported that most of their adaptation occurred during early implementation and implementation phase, and McCarthy *et al.* [18], reported most adaptations occurred during the mid-implementation phase.

Adaptations were documented through multiple methods including real-time tracking, reminder emails, team meetings, and interviews, aligning with the methodology employed by McCreight *et al.* [17]. Similar strategies, including real-time tracking and interviews, were also utilized by Rabin *et al.* [13]. Additionally, Reyes *et al.* captured adaptations through meeting notes, a method consistent with our approach. In our study, the majority of adaptations were documented through emails and meetings, collectively accounting for 21 adaptations. While, meetings served as a primary documentation method, capturing 13 adaptations, emails recorded seven adaptations. Furthermore, there were 22 adaptations that were captured by multiple methods indicating that Holtrop *et al.* [20]. specifically discussed the importance of using multiple methods to capture adaptation, indicating that not only the number of captured adaptations increases with multiple methods but the level of understanding of the given adaptation as well (i.e. multiple methods can be triangulated to fully appreciate what was changed, why, and with what impact).

The most commonly categorized adaptation element was “Other” (*n* = 19), which encompassed modifications related to the intervention materials and content. This distribution suggests that early-stage modification were largely directed toward optimizing intervention delivery and study infrastructure prior to full-scale implementation. Additionally, this may suggest that a possible opportunity to expand response categories for each domain. As with McCreight *et al.* [17]. and McCarthy *et al.* [18], the majority of adaptations in our study were driven by pragmatic or practical considerations (*n* = 22). However, Reyes *et al.* [19], reports that most of their adaptations were guided by feedback or suggestions.

We assessed the impact of adaptations on a diverse set of RE-AIM outcomes using a structured team-based reflection process. While most adaptations had neutral impact, several were clearly beneficial—e.g. centralizing FIT procurement to overcome COVID-related supply chain issues. Negative outcomes were rare, reinforcing prior findings that adaptation need not compromise implementation if carefully managed [21]. Assessment of the impact of adaptations is the most underdeveloped area in adaptation documentation. The methodology used in this paper is relatively novel and provides an example for the systematic assessment of adaptations. Future studies should refine how adaptation impact is assessed including exploring more objectives way of documenting adaptation impact. A recent paper by Aschbrenner et al., which was published after data collection and analysis was completed for our study, discusses key considerations (not results) for the assessment of adaptations and can provide as a basis for future assessment of adaptations [7].

This study has several notable strengths. Among them was the ability to document adaptations longitudinally—pre-implementation, implementation, and sustainment using a framework-guided approach by combining the strength of FRAME and RE-AIM. Multiple data sources, including emails and meeting records, were utilized to comprehensively capture adaptation activity allowing for more comprehensive capture of adaptations and more in depth understanding of the adaptations’ characteristics. Importantly, we were also able to assess the perceived impact of each adaptation on implementation and effectiveness outcomes. Each adaptation was categorized based on its observed impact: increased, decreased, no major change, unknown, or not applicable.

Adaptation impact documentation is its early stage and many questions remain specially for adaptations that are made based on our implementation experience or change in context. While capturing adaptations provided feasible, comprehensive, and informative, there were limitations in the application of our strategy. In an effort to reduce burden, the participating CHCs were not sent regular email prompts to report adaptations. Instead, the CHCs were asked to report adaptations when prompted during scheduled team meetings led by the implementation science team. As a result, it is likely that unplanned and planned adaptations may have gone unreported, particularly given the lack of direct and continuous engaged with the CHCs personnel. While we attempted to mitigate these issues through regular check-ins and document reviews, future work may benefit from embedding adaptation tracking directly into study workflows and incentivizing real-time reporting. Additionally, in this specific study we only used two levels to document adaptations. Future studies could consider the levels of change in a more sophisticated way, including analyzing adaptations from a different angle (i.e. explanatory format to demonstrate changes occurring to the setting and format).

Additionally, adaptation impact was recorded based on perception of those most knowledgeable about outcomes instead of objective, systematically collected data. As noted earlier, this area is still under development and needs more attention. Specifically, future studies should aim to develop a more robust, longitudinal (going through sustainment), and objective design and methods for purposefully evaluating the impact of adaptations and consider using established frameworks for adaptation impact assessment [7, 22].

## Conclusion

Our findings highlight the importance of proactively documenting adaptations across all stages of implementation. Our multi-method, framework-driven approach can serve as a model for other implementation efforts, particularly in safety-net and community-based healthcare systems. By understanding when, why, and how adaptations occur, researchers and practitioners can enhance intervention fit, address context-specific challenges, and improve implementation fidelity and sustainability.

## Funding sources

This study was funded by the National Cancer Institute (grant number UH3 CA233314). The funders had no role in the design and conduct of the study; collection, management, analysis, and interpretation of the data; preparation, review, or approval of the manuscript; or decision to submit the manuscript for publication.

## Supplementary Material

ibag072_Supplementary_Data

## Data Availability

De-identified data from this study are not available in a public archive. De-identified data from this study will be made available (as allowable according to institutional IRB standards) by emailing the corresponding author.

## Welfare of animals

This article does not contain any studies with animals performed by any of the authors.

## Artificial Intelligence

Artificial intelligence was not used in the development of this manuscript.
