# Supplementary material for: Multi-method, longitudinal documentation of adaptations and their impact in the Accelerating Colorectal Cancer Screening and Follow-up through Implementation Science San Diego colorectal cancer screening implementation study
Source: Transl Behav Med. 2026 Oct 6;16(1):ibag072. doi: 10.1093/tbm/ibag072 (PMC13641175; doi:10.1093/tbm/ibag072)
Supplement: Supplementary file 1 [file ibag072_supplementary_data.docx]

| **Appendix 1. Detailed list and characteristics of adaptation documented in the ACCSIS study** | | | | | | | | | |
| --- | --- | --- | --- | --- | --- | --- | --- | --- | --- |
| **Adaptation Title** | **Adaptation Brief Summary** | **What element was changed?** | **What was the type of change?** | **Which core component is impacted by the change?** | **Who was responsible for initiating this adaptation?** | **What was the adaptation based on?** | **Was the adaptation due to the COVID-19 pandemic?** | **Why was this adaptation made?** | **What was the impact of this adaptation?** |
| Include all patients in the intervention clinics in the sample size | All patients at the three intervention clinics will only receive a FIT from our study | The format | Loosening the structure or protocol | Mailed FIT process | Entire or most of team | Based on pragmatic/  practical considerations  (for example, “this is the only way it would work”) | No | To enhance the impact or success of the intervention for all or important subgroups (effectiveness)  To make the intervention delivered more consistently; to better fit the CHC/clinic, clinician needs, patient flow or EHR; for practical reasons (implementation) | Quality of care or other outcomes (effectiveness) *No Major Changes*  Consistent delivery of quality care or costs (implementation) *No Major Changes*  Reimbursement or financial implications for the practice (implementation) *No Major Changes* |
| Revised patient reminder schedule | Includes an additional text message | The format | Loosening the structure or protocol | Mailed FIT process | HQP | Additional funds availability to increase intensity* | No | To enhance the impact or success of the intervention for all or important subgroups (effectiveness)  To save money or other resources (implementation) | Number or type of patients engaged (reach) *Do not know*  Consistent delivery of quality care or costs (implementation) *Do not know*  Reimbursement or financial implications for the practice (implementation) *Do not know* |
| Modification in mailed FIT outreach | All participating CHCs agreed to collaborate with a third-party vendor to deliver the mailed FIT intervention | How the intervention is presented | Substituting for a component | Mailed FIT process | Entire or most of team | Based on pragmatic/  practical considerations (for example, “this is the only way it would work”) | No | To make the intervention delivered more consistently; to better fit the CHC/clinic, clinician needs, patient flow or EHR; for practical reasons (implementation) | Consistent delivery of quality care or costs (implementation) *Not applicable*  Reimbursement or financial implications for the practice (implementation) *Not applicable* |
| Modification in mailed FIT outreach | The study team approved the use of a third-party vendor to disseminate the mailed FIT intervention component to study participants instead of HQP doing this in-house | How the intervention is presented | Integrating with other programs we are doing | Mailed FIT process | Entire or most of team | Based on pragmatic/  practical considerations  (for example, “this is the only way it would work”) | No | To make the intervention delivered more consistently; to better fit the CHC/clinic, clinician needs, patient flow or EHR; for practical reasons (implementation) | Consistent delivery of quality care or costs (implementation) *Do not know*  Reimbursement or financial implications for the practice (implementation) *Do not know*  Efficiency (getting more done faster or with less resources)  *Increase* |
| Distribution of mailed FIT invitations | CHC request 3 waves in year one | The format | Loosening the structure or protocol | Mailed FIT process | Entire or most of team | Based on pragmatic/  practical considerations  (for example, “this is the only way it would work”) | No | To make the intervention delivered more consistently; to better fit the CHC/clinic, clinician needs, patient flow or EHR; for practical reasons (implementation) | Consistent delivery of quality care or costs (implementation) *No Major Change*  Reimbursement or financial implications for the practice (implementation) *No Major Change*  Efficiency (getting more done faster or with less resources)  *Increase* |
| Changes to mailed FIT invitation material | Revise primer in Spanish | Other | Otherwise changing the intervention | Mailed FIT process | CHC or Individual Clinic | Based on feedback or suggestions (Practice Facilitator/  coach or other) | No | To increase the number or type of patients contacted (reach) | Number or type of patients engaged (reach) *No Major Change* |
| Changes to mailed FIT invitation material | Allows opportunity to highlight partnership between HQP and CHCs | Other | Otherwise changing the intervention | Mailed FIT process | CHC or Individual Clinic | Based on our knowledge or experience of working with patients | No | To enhance the impact or success of the intervention for all or important subgroups (effectiveness)  To make the intervention delivered more consistently; to better fit the CHC/clinic, clinician needs, patient flow or EHR; for practical reasons (implementation) | Quality of care or other outcomes (effectiveness) *Do not know*  Consistent delivery of quality care or costs (implementation) *No Major Change*  Reimbursement or financial implications for the practice (implementation) *No Major Change* |
| Changes to mailed FIT invitation material | Revise primer in Spanish | Other | Otherwise changing the intervention | Mailed FIT process | CHC or Individual Clinic | Based on feedback or suggestions (Practice Facilitator/  coach or other) | No | To increase the number or type of patients contacted (reach) | Number or type of patients engaged (reach) *Do not know* |
| COVID-19 Messaging | Development of COVID-19 messaging (postcard) to include in the FIT kit package | Other | Extending a component | Mailed FIT process | Entire or most of team | Based on our knowledge or experience of working with patients | Yes | To enhance the impact or success of the intervention for all or important subgroups (effectiveness)  To respond to external pressures or policy (implementation) | Quality of care or other outcomes (effectiveness) *Do not know*  Consistent delivery of quality care or costs (implementation) *No Major Change* |
| Changes to mailed FIT invitation material | Revise primer | Other | Otherwise changing the intervention | Mailed FIT process | Research Team | Based on our knowledge or experience of working with patients | No | To enhance the impact or success of the intervention for all or important subgroups (effectiveness) | Quality of care or other outcomes (effectiveness) *Do not know* |
| Changes to mailed FIT invitation material | Underline/  emphasize | Other | Otherwise changing the intervention | Mailed FIT process | CHC or Individual Clinic | Based on our knowledge or experience of working with patients | No | To enhance the impact or success of the intervention for all or important subgroups (effectiveness) | Quality of care or other outcomes (effectiveness) *Do not know* |
| Mailed FIT: Eligibility Criteria | Revise eligibility criteria | Other | Condensing a component | Mailed FIT process | Entire or most of team  Research Team | Based on feedback or suggestions (Practice Facilitator/  coach or other) | No | To make the intervention delivered more consistently; to better fit the CHC/clinic, clinician needs, patient flow or EHR; for practical reasons (implementation) | Consistent delivery of quality care or costs (implementation) *No Major Change*  Reimbursement or financial implications for the practice (implementation) *No Major Change* |
| Usual Care Assessment | Modify Usual Care Assessment | The format | Extending a component | Usual Care Assessment Protocol | Research Team | Based on feedback or suggestions (Practice Facilitator/  coach or other) | Yes | To enhance the impact or success of the intervention for all or important subgroups (effectiveness) | Quality of care or other outcomes (effectiveness) *No Major Change* |
| Abnormal FIT Follow-up Training | Care Coordination Training: virtual trainings for care coordinators | The setting | Substituting for a component | Care coordination process | Entire or most of team | Based on pragmatic/  practical considerations (for example "this is the only way it would work") | Yes | To make the intervention delivered more consistently; to better fit the CHC/clinic, clinician needs, patient flow or EHR; for practical reasons (implementation), To respond to external pressures or policy (implementation) | Consistent delivery of quality care or costs (implementation) *No Major Change*  Reimbursement or financial implications for the practice (implementation) *No Major Change* |
| Abnormal FIT Follow-up: Train-the-Trainer Model | Train-the-trainer model was adopted to accommodate staff turnover and facilitate onboarding | The setting | Loosening the structure or protocol | Care coordination process | Entire or most of team | Based on pragmatic/practical considerations (for example "this is the only way it would work") | Yes | To make the intervention delivered more consistently; to better fit the CHC/clinic, clinician needs, patient flow or EHR; for practical reasons (implementation)  To respond to external pressures or policy (implementation) | Consistent delivery of quality care or costs (implementation) *No Major Change*  Reimbursement or financial implications for the practice (implementation) *No Major Change*  Efficiency (getting more done faster or with less resources)  *Increase* |
| FIT Supply | Securing FITs have been challenging. HQP used established contacts at the contract labs to assist with getting bulk FIT supplies | Other | How the intervention is delivered in the study* | Mailed FIT process | HQP  CHC or Individual Clinic | Based on pragmatic/  practical considerations (for example "this is the only way it would work") | Yes | To respond to external pressures or policy | Reimbursement or financial implications for the practice *Increase*  Efficiency (getting more done faster or with less resources)  *Increase* |
| CHC Staff Member | Coordinator at CHC resigned | Personnel involved | How the intervention is delivered in the study* | Change in personnel | CHC or Individual Clinic | Based on pragmatic/  practical considerations (for example "this is the only way it would work") | No | to respond to internal change (i.e., staff change, site closures, CHC preference, etc.)* (implementation) | Consistent delivery of quality care or costs (implementation) *No Major Changes* |
| Colorectal Cancer Screening text message campaign | Automated text messaging Colorectal Cancer Screening campaign for participating CHCs | Other | Integrating with other programs we are doing | Mailed FIT process | HQP  Other | Based on pragmatic/  practical considerations (for example "this is the only way it would work") | No | To make the intervention delivered more consistently; to better fit the CHC/clinic, clinician needs, patient flow or EHR; for practical reasons (implementation) | Consistent delivery of quality care or costs (implementation) *No Major Change*  Reimbursement or financial implications for the practice (implementation) *No Major Change* |
| FIT Order | Two participating CHCs decided to place the FIT order after an eligible patient returned a completed FIT | Other | How the intervention is delivered in the study* | Mailed FIT process | CHC or Individual Clinic | Based on feedback or suggestions (Practice Facilitator/  coach or other) | No | To save money or other resources (implementation) | Consistent delivery of quality care or costs (implementation) *No Major Change*  Maintenance or sustainability of the intervention in the practice (maintenance) *Increase*  Reimbursement or financial implications for the practice *Increase*  Efficiency (getting more done faster or with less resources)  *Increase* |
| Usual Care Site Closure | A usual care site at one participating CHC closed | The setting | Study related changes* | Change in participating sites | CHC or Individual Clinic | Based on pragmatic/  practical considerations (for example "this is the only way it would work") | No | to respond to internal change (i.e., staff change, site closures, CHC preference, etc.)* (implementation) | Consistent delivery of quality care or costs (implementation) *No Major Changes* |
| Patient Sample Selection | A participating CHC obtained approval by the MPIs to scrub their own data | The target population | Study related changes* | Data management | CHC or Individual Clinic | Based on feedback or suggestions (Practice Facilitator/  coach or other) | No | To make the intervention delivered more consistently; to better fit the CHC/clinic, clinician needs, patient flow or EHR; for practical reasons (implementation) | Consistent delivery of quality care or costs (implementation) *No Major Change*  Reimbursement or financial implications for the practice (implementation) *No Major Change* |
| Changes to mailed FIT invitation material | Changes to the FIT kit instructions (brand A) in English and Spanish | Other | Otherwise changing the intervention | Mailed FIT process | Research Team  HQP | Based on QI data, summary information or results | No | To make the intervention delivered more consistently; to better fit the CHC/clinic, clinician needs, patient flow or EHR; for practical reasons (implementation) | Consistent delivery of quality care or costs (implementation) *No Major Change*  Reimbursement or financial implications for the practice (implementation) *No Major Change* |
| Changes to mailed FIT invitation material | Changes to the FIT kit instructions (brand B) in English and Spanish | Other | Otherwise changing the intervention | Mailed FIT process | Research Team  HQP | Based on QI data, summary information or results | No | To make the intervention delivered more consistently; to better fit the CHC/clinic, clinician needs, patient flow or EHR; for practical reasons (implementation) | Consistent delivery of quality care or costs (implementation) *No Major Change*  Reimbursement or financial implications for the practice (implementation) *No Major Change* |
| Changes to Care Coordination materials | Materials were modified to accurately capture patient refusal of care coordination intervention and/or colonoscopy | The format | Otherwise changing the intervention | Care coordination process  Data management | Research Team | Based on QI data, summary information or results (new theme) | No | To make the intervention delivered more consistently; to better fit the CHC/clinic, clinician needs, patient flow or EHR; for practical reasons (implementation) | Consistent delivery of quality care or costs (implementation) *No Major Change*  Reimbursement or financial implications for the practice (implementation) *No Major Change* |
| Usual Care Assessment | Modify Usual Care Assessment | The format | Extending a component | Usual Care Assessment Protocol | Research Team | Based on feedback or suggestions (Practice Facilitator/  coach or other) | No | To enhance the impact or success of the intervention for all or important subgroups (effectiveness) | Quality of care or other outcomes (effectiveness) *No Major Changes* |
| Mailing of Normal Results Letters | No longer provided by the study staff | Personnel involved | Removing a component  Substituting for a component | Mailed FIT process | Entire or most of team | Based on our knowledge or experience of working with patients  Based on feedback or suggestions (Practice Facilitator/  coach or other) | No | To make the intervention delivered more consistently; to better fit the CHC/clinic, clinician needs, patient flow or EHR; for practical reasons (implementation) | Consistent delivery of quality care or costs (implementation) *Do not know*  Reimbursement or financial implications for the practice (implementation) *Do not know* |
| CHC withdrawal | CHC submitted notice of termination | The setting | Study related changes* | Change in participating sites* | CHC or Individual Clinic | Based on pragmatic/  practical considerations (for example "this is the only way it would work") | No | To respond to external pressures or policy (implementation) | Number or type of patients engaged (reach) *Decrease*  Consistent delivery of quality care or costs (implementation) *Not applicable* |
| Changes to mailed FIT material | Replacing the envelopes included in the FIT kit envelopes with a new yellow envelope | Other | Otherwise changing the intervention | Mailed FIT process | HQP  FIT vendor | Based on pragmatic/  practical considerations (for example "this is the only way it would work") | Yes | To make the intervention delivered more consistently; to better fit the CHC/clinic, clinician needs, patient flow or EHR; for practical reasons (implementation) | Consistent delivery of quality care or costs (implementation) *No Major Change*  Reimbursement or financial implications for the practice (implementation) *No Major Change* |
| CHC withdrawal | CHC submitted notice of termination | The target population | Study related changes* | Change in participating sites* | CHC or Individual Clinic | Based on our vision or values  Based on our knowledge or experience of working with patients  Based on pragmatic/  practical considerations (for example "this is the only way it would work") | No | To increase the number or type of patients contacted (reach)  To enhance the impact or success of the intervention for all or important subgroups (effectiveness)  To make it possible to involve more teams, team members or staff (adoption)  To make the intervention delivered more consistently; to better fit the CHC/clinic, clinician needs, patient flow or EHR; for practical reasons (implementation)  To institutionalize or sustain the intervention (maintenance)* | Number or type of patients engaged (reach) *Decrease*  Quality of care or other outcomes (effectiveness) *No Major Change*  Consistent delivery of quality care or costs (implementation) *No Major Change*  Reimbursement or financial implications for the practice (implementation) *No Major Change*  Maintenance or sustainability of the intervention in the practice (maintenance) *No Major Change*  Maintenance or sustainability of the patient within the intervention (maintenance) *No Major Change* |
| CHC Staff Member | Coordinator at CHC resigned | Personnel involved | How the intervention is delivered in the study* | Change in personnel* | CHC or Individual Clinic | Based on pragmatic/  practical considerations (for example "this is the only way it would work") | No | to respond to internal change (i.e., staff change, site closures, CHC preference, etc.)* (implementation) | Consistent delivery of quality care or costs (implementation) *No Major Changes* |
| Study Notation in Lab Notes/EHR | One CHC is having their laboratory staff add a note in their EHR that says "ACCSIS Study" | Other | How the intervention is delivered in the study* | Mailed FIT process | CHC or Individual Clinic | Based on pragmatic/  practical considerations (for example "this is the only way it would work") | No | to respond to internal change (i.e., staff change, site closures, CHC preference, etc.)* (implementation) | Consistent delivery of quality care or costs (implementation) *No Major Changes* |
| Patient sample selection | After reviewing the eligible sample and limiting it to age 50 -74 with an expanding date range, patients from a wave were found to no longer be eligible after a Primer was sent | Other | Otherwise changing the intervention | Mailed FIT process | Research Team  CHC or Individual Clinic | Based on our knowledge or experience of working with patients  Based on pragmatic/  practical considerations (for example "this is the only way it would work") | No | To make the intervention delivered more consistently; to better fit the CHC/clinic, clinician needs, patient flow or EHR; for practical reasons (implementation) | Number or type of patients engaged (reach) *Increase* |
| CHC clinic site changes | Change intervention sites | The setting | Study related changes* | Change in participating sites | CHC or Individual Clinic | Based on pragmatic/  practical considerations (for example "this is the only way it would work") | No | to respond to internal change (i.e., staff change, site closures, CHC preference, etc.)* (implementation) | Consistent delivery of quality care or costs (implementation) *No Major Changes* |
| CHC Staff Changes | Two staff members joined the team and will be focused on care coordination | Personnel involved | How the intervention is delivered in the study* | Change in personnel* | CHC or Individual Clinic | Based on pragmatic/  practical considerations (for example "this is the only way it would work") | No | to respond to internal change (i.e., staff change, site closures, CHC preference, etc.)* (implementation) | Consistent delivery of quality care or costs (implementation) *No Major Changes* |
| CHC Staff Changes | Two staff changes: Staff were on the ACCSIS care coordination / patient navigation team | Personnel involved | How the intervention is delivered in the study* | Change in personnel* | CHC or Individual Clinic | Based on pragmatic/  practical considerations (for example "this is the only way it would work") | No | to respond to internal change (i.e., staff change, site closures, CHC preference, etc.)* (implementation) | Consistent delivery of quality care or costs (implementation) *No Major Changes* |
| Third party vendor reminder error | Additional unplanned reminders to patients | How the intervention is presented | Otherwise changing the intervention | Mailed FIT process | FIT Vendor | Error* | No | To make the intervention delivered more consistently; to better fit the CHC/clinic, clinician needs, patient flow or EHR; for practical reasons (implementation) | Consistent delivery of quality care or costs (implementation) *No Major Change*  Reimbursement or financial implications for the practice (implementation) *No Major Change* |
| Addition of test to Care Coordination Logs | Modification of the include "Cologuard" in the drop-down option for test type | Other | Study related changes* | Data management | CHC or Individual Clinic | Based on pragmatic/  practical considerations (for example "this is the only way it would work") | No | To respond to external pressures or policy (implementation)  to respond to internal change (i.e., staff change, site closures, CHC preference, etc.)* (implementation) | Consistent delivery of quality care or costs (implementation) *No Major Changes* |
| Third party vendor reminder error | Delay in reminders to patients | How the intervention is presented | Otherwise changing the intervention | Mailed FIT process | FIT Vendor | Error* | No | To make the intervention delivered more consistently; to better fit the CHC/clinic, clinician needs, patient flow or EHR; for practical reasons (implementation) | Consistent delivery of quality care or costs (implementation) *No Major Change*  Reimbursement or financial implications for the practice (implementation) *No Major Change* |
| Third party vendor error | Failing to distribute FIT kits by mail to a small sample of patients | The target population  How the intervention is presented | Otherwise changing the intervention | Mailed FIT process | FIT Vendor | Error* | No | To make the intervention delivered more consistently; to better fit the CHC/clinic, clinician needs, patient flow or EHR; for practical reasons (implementation) | Consistent delivery of quality care or costs (implementation) *No Major Change*  Reimbursement or financial implications for the practice (implementation) *No Major Change* |
| Return Address Change for Mailed FIT Materials | HQP moved to a permanently remote working environment, so PO Box established | Other | Study related changes* | Change in participating sites* | HQP | Based on pragmatic/  practical considerations (for example "this is the only way it would work") | Yes | To respond to external pressures or policy  (implementation) | Consistent delivery of quality care or costs (implementation) *No Major Changes* |
| Missing Data | Study statistician identified patients with abnormal FIT results in the dataset, but were not on the abnormal FIT logs | Other | How the intervention is delivered in the study* | Care coordination process  Data management | Research Team | Based on QI data, summary information or results | No | To increase the number or type of patients contacted (reach) | Number or type of patients engaged (reach) *No Major Change* |
| Change in MPIship | Upon receiving approval from NCI, a new PI was identified | Personnel involved | Study related changes* | Change in personnel* | Research team | Based on pragmatic/  practical considerations (for example "this is the only way it would work") | No | To respond to external pressures or policy  (implementation) | Consistent delivery of quality care or costs (implementation) *No Major Changes* |
| Care Coordination Protocol Modified | Revisions were made to the Key Activities Tracking Log and Instructions | Other | Otherwise changing the intervention | Care coordination process | Entire or most of team  HQP | Based on QI data, summary information or results | No | To make the intervention delivered more consistently; to better fit the CHC/clinic, clinician needs, patient flow or EHR; for practical reasons (implementation) | Consistent delivery of quality care or costs (implementation) *No Major Change*  Reimbursement or financial implications for the practice (implementation) *No Major Change* |
| Patient sample selection | Conduct a repeat data pull of Year 2 patients in order to bring our Year 2 invitations up to 5000 | The setting  The format | Changing the order of components | Mailed FIT process | Entire or most of team | Based on QI data, summary information or results | No | To increase the number or type of patients contacted (reach)  To enhance the impact or success of the intervention for all or important subgroups (effectiveness) | Number or type of patients engaged (reach) *Do Not Know*  Quality of care or other outcomes (effectiveness) *No Major Change* |

*New theme

**Appendix 2: Evidence-based intervention workflow diagram**

**Appendix 3: Adaptations Tracking Tool**

| Field Name | Scales/Notes |
| --- | --- |
| 1. Person entering adaptation information into database | *This is typically the implementation coordinator* |
| 2. Site(s) where adaptation is relevant | ☐ [Name of community health center (CHC) 1]  ☐ [Name of CHC 2]  ☐ [Name of CHC 3]  ☐ [Name of CHC 4]  ☐ [Non-CHC adaptation]  *Select all that apply* |
| Please specify clinic site(s) | Name of CHC 1  ☐ [All Clinics]  ☐ [Name of clinic site(s) 1]  ☐ [Name of clinic site(s) 2]  ☐ [Name of clinic site(s) 3]  Name of CHC 2  ☐ [All Clinics]  ☐ [Name of clinic site(s) 1]  ☐ [Name of clinic site(s) 2]  ☐ [Name of clinic site(s) 3]  Name of CHC 3  ☐ [All Clinics]  ☐ [Name of clinic site(s) 1]  ☐ [Name of clinic site(s) 2]  ☐ [Name of clinic site(s) 3]    Name of CHC 4  ☐ [All Clinics]  ☐ [Name of clinic site(s) 1]  ☐ [Name of clinic site(s) 2]  ☐ [Name of clinic site(s) 3] |
| **Adaptation Information** | |
| 3. Date when the adaptation is entered into database | *Name of implementation coordinator.* |
| 4. Date when the adaptation was first enacted or implemented | *Format: DD/MM/YYYY*  *Please give your best estimate* |
| 5. Name of the adaptation | *e.g. change in recruitment* |
| 6. How was the information on the adaptation documented? | *Documentation of adaptation / i.e. meeting, email, notes, interviews* |
| 7. Brief description of the adaptation that was made | *Try to keep it 1-2 sentences long, but provide enough context that it stands alone / e.g. recruitment criteria was changed to include all patients with XX code as well.* |
| 8. Who informed you about the adaptation? | *e.g. leadership, coordinator, program manager, medical assistant 3rd party vendor – delivery of mailed FIT intervention, research staff* |
| 9. Position of the person providing information about the adaptation | ☐ Research Staff  ☐ Hub  ☐ Physician  ☐ Clinic Coordinator  ☐ Lab Manager  ☐ 3^rd^ party vendor – delivery of mailed FIT intervention  ☐ Other  *Position of informant* |
| If other, please specify position |  |
| **Changes Made by Adaptation** | |
| 10. Which of the following elements was primarily changed as part of the adaptation? | ☐ The setting  ☐ The format  ☐ Personnel involved  ☐ The target population  ☐ How the intervention is presented  ☐ Other  *Check all that apply* |
| If other, please specify |  |
| 11. What type of change happened? | ☐ Tailoring to individual  ☐ Adding a component  ☐ Removing a component  ☐ Condensing a component  ☐ Extending a component  ☐ Substituting for a component  ☐ Changing the order of components  ☐ Integrating with other programs we are doing  ☐ Repeating a component  ☐ Loosening the structure of protocol  ☐ Otherwise changing the intervention  *Check all that apply* |
| If otherwise changing the intervention, please specify |  |
| 12. Study core components impacted by the adaptation: | ☐ Mailed Fecal Immunochemical Test (FIT) process  ☐ Care coordination process  ☐ Research logistics (IRB, language translation, incentives)  ☐ Data collection and analysis process  ☐ Other  *Check all that apply* |
| If other, please specify |  |
| 13. Who was primarily responsible for initiating this | ☐ Entire or most of team  ☐ Research team  ☐ Hub  ☐ CHC or Individual Clinic  ☐ Other  *People who initiated the adaptation*  *Check all that apply* |
| If other, please specify |  |
| 14. At which of the following points in the project was this change FIRST made? | ☐ Pre-implementation (before Mailed FIT and Care Coordination starts)  ☐ Implementation (during Mailed FIT and Care Coordination starts)  ☐ Sustainment (after Mailed FIT and Care Coordination starts) |

| 15. What was the change based on? | ☐ Based on our vision or values  ☐ Based on a framework  ☐ Based on our knowledge or experiences of working with patients  ☐ Based on patient knowledge or experiences  ☐ Based on QI data, summary information or results  ☐ Based on pragmatic/practical considerations (for example, “this is the only way it would work”)  ☐ Based on financial incentives/payment  ☐ Based on feedback or suggestions (practice facilitators/coach or other)  ☐ Other  *Check all that apply* |
| --- | --- |
| If other, please specify |  |
| Was this change related to the COVID-19 pandemic? | ☐ Yes  ☐ No |
| If yes, please explain |  |
| 16. Why was this change made? | ☐ To increase the number or type of patients engaged (reach)  ☐ To enhance the impact or success of the intervention for all or important subgroups (effectiveness)  ☐ To make it possible to involve more teams, team members or staff (adoption)  ☐ To make the intervention delivered more consistently; to better fit the CHC/clinic, clinician needs, patient flow or Electronic Health Record; for practical reasons (implementation)  ☐ To institutionalize or sustain the intervention (maintenance)  ☐ To respond to external pressures or policy  ☐ To save money or other resources (implementation)  ☒ Other  *Reasoning behind the adaptation. Check all that apply* |
| If other, please specify |  |
| 17. What kind of changes did you see as a result of this adaptation? | |
| a. Number or type of patients engaged (reach) | ☐ Too early to know  ☐ Increase  ☐ Decrease  ☐ No Major Change |
| b. Quality of care or other resources (effectiveness) | ☐ Too early to know  ☐ Increase  ☐ Decrease  ☐ No Major Change |
| c. Participation by teams or staff (adoption) | ☐ Too early to know  ☐ Increase  ☐ Decrease  ☐ No Major Change |
| d. Consistent delivery of quality care or costs (implementation) | ☐ Too early to know  ☐ Increase  ☐ Decrease  ☐ No Major Change |
| e. Maintenance or sustainability of the intervention in the practice (maintenance) | ☐ Too early to know  ☐ Increase  ☐ Decrease  ☐ No Major Change |
| f. Maintenance or sustainability of the patient within the practice (maintenance) | ☐ Too early to know  ☐ Increase  ☐ Decrease  ☐ No Major Change |
| g. Reimbursement or financial implications for the practice | ☐ Too early to know  ☐ Increase  ☐ Decrease  ☐ No Major Change |
| h. Efficiency (getting more done faster or with fewer resources) | ☐ Too early to know  ☐ Increase  ☐ Decrease  ☐ No Major Change |
| Other changes (specify) |  |
| 18. Whose need did the adaptation addressed? | ☐ Research team/Hub/3^rd^ party vendor – delivery of mailed FIT intervention  ☐ Community Health Center/Clinic  ☐ Patient  ☐ Other  *Check all that apply* |

| If other, please specify |  |
| --- | --- |
| Any additional comments on the adaptation? |  |
| Please include any supporting documents | *Accepted files include: PDF, Word, Excel, PowerPoint, JPG. As along as the file size is under 32 MB.* |

| **FOR INTERNAL COMPLETION BY IMPLEMENTATION SCIENCE TEAM MEMBERS ONLY** | |
| --- | --- |
| Did team member(s) validate this adaptation? | ☐ Yes, validation complete  ☐ No, not yet  ☐ Sort of, in progress |
| Enter date of validation |  |

**Appendix 4: Email Reminder Template**

Hello Team,

Just a gentle reminder, please send [Insert Name of Implementation Coordinator] any changes and modifications (adaptations) that you are aware of regarding how we deliver our study activities that occurred over the past approximately 2 weeks. These changes and modifications can vary across settings and might be made to processes, personnel, content, and format. Feel free to be inclusive in reporting these. **Please send a brief reply to this email to [Insert Name of Implementation Coordinator] with any changes and modifications by 5PM on Friday.**

By adaptations, we mean changes to the CRC screening interventions and implementation strategies compared to what was initially planned. Think about changes broadly and include those that happened pre-implementation and during implementation, those that were planned or unplanned, and those that were associated with the intervention and/or the implementation strategies.

**Appendix 5: Mid-Implementation and End-Implementation Interview Guides**

*The following interview items were selected to demonstrate relevance to adaptations.*

**Mid and End Implementation**

*Wording was changed depending if the interview was conducted mid-implementation / end-implementation

**ACCSIS Project: Mailed FIT Outreach**

***Interviewer:*** “The first of two major components of the ACCSIS project is the mailed FIT outreach. An abbreviated flowchart of the ACCSIS Mailed FIT Outreach component is displayed.” [*Share your screen and* *verbally review the flowchart below*]

1. What challenges have you encountered *to date / during the past 12 months beginning in 2023**, while implementing the mailed FIT outreach component of the ACCSIS program?

Probe: Please think about both internal (e.g. staffing) and external factors (e.g. COVID-19) that influenced the implementation of the mailed FIT component of the ACCSIS program.

***Interviewer:*** *“*We expect that as the context is changing, changes might also happen to the program.”

2. What changes did you make to the mailed FIT outreach program activities, flow, or other aspects to address these challenges or for other reasons?
   a. Were there any key changes you made to the CRC screening program? How it is delivered at your clinic/CHC?
   b. Why were these changes made?
   c. When in the implementation process were these changes made?
   d. What impact did these changes have on the implementation of the program and/or the outcomes?

**ACCSIS Project: Abnormal FIT Follow-up**

***Interviewer:*** “The second major component of the ACCSIS project is the Abnormal FIT Follow-up. An abbreviated flowchart of the ACCSIS Abnormal FIT Follow-up component is displayed.” [*Share your screen and* *verbally review the flowchart below*].

1. What challenges have you encountered to date while implementing the abnormal FIT follow-up component of the ACCSIS program?

Probe: Please think about both internal and external factors that influenced the implementation of the abnormal FIT follow-up component of the ACCSIS program.

2. Describe challenges, if any, related to patients having access to colonoscopy and abnormal FIT follow-up.

***Interviewer:*** *“*We expect that as the context is changing, changes might also happen to the program.”

3. What changes did you make to the abnormal FIT follow-up program activities, flow, or other aspects to address these challenges or for other reasons?
   a. Were there any key changes you made to the CRC screening program? How it is delivered at your clinic/CHC?
   b. Why were these changes made?
   c. When in the implementation process were these changes made?
   d. What impact did these changes have on the implementation of the program and/or the outcomes?

**ACCSIS Project: Sustainment**

Keeping the hub-spoke model in mind, please describe any aspects of this model that created **challenges** for implementing the ACCSIS program at your CHC.
